# Comparative analysis of mycobacterial NADH pyrophosphatase isoforms reveals a novel mechanism for isoniazid and ethionamide inactivation

**DOI:** 10.1111/j.1365-2958.2011.07892.x

**Published:** 2011-11-03

**Authors:** Xu-De Wang, Jing Gu, Ting Wang, Li-Jun Bi, Zhi-Ping Zhang, Zong-Qiang Cui, Hong-Ping Wei, Jiao-Yu Deng, Xian-En Zhang

**Affiliations:** 1 State Key Laboratory of VirologyWuhan 430071, China; 2 Key Laboratory of Agricultural and Environmental Microbiology, Wuhan Institute of Virology, Chinese Academy of SciencesWuhan 430071, China; 3 Graduate school, Chinese Academy of ScienceBeijing 100039, China; 4 National Laboratory of Biomacromolecules, Institute of Biophysics, Chinese Academy of SciencesBeijing 100101, China

## Abstract

NADH pyrophosphatase (NudC) catalyses the hydrolysis of NAD(H) to AMP and NMN(H) [nicotinamide mononucleotide (reduced form)]. NudC multiple sequence alignment reveals that homologues from most *Mycobacterium tuberculosis* isolates, but not other mycobacterial species, have a polymorphism at the highly conserved residue 237. To elucidate the functional significance of this polymorphism, comparative analyses were performed using representative NudC isoforms from *M. tuberculosis* H37Rv (NudC_Rv_) and *M. bovis* BCG (NudC_BCG_). Biochemical analysis showed that the P237Q polymorphism prevents dimer formation, and results in a loss of enzymatic activity. Importantly, NudC_BCG_ was found to degrade the active forms of isoniazid (INH), INH-NAD and ethionamide (ETH), ETH-NAD. Consequently, overexpression of NudC_BCG_ in *Mycobacterium smegmatis* mc^2^155 and *M. bovis* BCG resulted in a high level of resistance to both INH and ETH. Further genetic studies showed that deletion of the *nudC* gene in *M. smegmatis* mc^2^155 and *M. bovis* BCG resulted in increased susceptibility to INH and ETH. Moreover, inactivation of NudC in both strains caused a defect in drug tolerance phenotype for both drugs in exposure assays. Taken together, these data suggest that mycobacterial NudC plays an important role in the inactivation of INH and ETH.

## Introduction

World Health Organization estimates suggest that *Mycobacterium tuberculosis* kills over 1 million people each year and causes over 8 million incident cases of tuberculosis (TB). The ever increasing incidence of drug-resistant TB further compounds this global health crisis. By the year 2007, there were approximately 0.5 million reported cases of multidrug-resistant TB. Moreover, by November 2009, 57 countries and territories had reported at least one case of extensively drug-resistant TB (World Health Organization, 2009). To counter this increasing threat of drug resistance, it is critical to understand fundamental aspects of TB-related biology. Such studies will not only uncover cellular mediators of susceptibility and resistance to existing anti-TB drugs, but will also provide new drug targets for the design of novel therapeutic agents.

Isoniazid (INH) was first described in the early 1900s, and since 1952 has prevailed as one of the most potent and widely administered anti-TB drugs (Bernstein *et al*., 1952; Fox, 1952). Over the last few decades, several genetic and biochemical studies have delineated the series of events involved in INH action. Upon entry into the cell, the hydrazide group of INH is activated by catalase-peroxidase (KatG) to generate an isonicotinic acyl radical. Once formed, this radical spontaneously reacts with NAD(H) at the 4th position of the nicotinamide ring, yielding an INH-NAD adduct (Zhang *et al*., 1992; Johnsson and Schultz, 1994; Rozwarski *et al*., 1998; Wilming and Johnsson, 1999). This adduct acts as a slow-onset, tight-binding inhibitor of the NADH-dependent enoyl-ACP reductase (InhA) of the mycobacterial fatty acid synthase II (Banerjee *et al*., 1994; Rozwarski *et al*., 1998; Wilming and Johnsson, 1999; Rawat *et al*., 2003). Competitive inhibition of NADH binding by INH-NAD results in cessation of mycolic acid biosynthesis and cellular lysis (Takayama *et al*., 1972; Vilchèze *et al*., 2000).

INH-resistant mutants of *M. tuberculosis* were recognized within the first year of the clinical use of INH as an anti-tubercular drug (Middlebrook and Cohn, 1953). Several mechanisms of INH resistance have since been described. These include failure to form the INH-NAD adduct due to loss of function mutations in *katG* (Heym and Cole, 1992; Zhang *et al*., 1992; Heym *et al*., 1995); failure of the INH-NAD adduct to associate with the target due to active-site mutations in *inhA* (Banerjee *et al*., 1994; Vilchèze *et al*., 2006); out-competition of the INH-NAD adduct with excessive cytoplasmic levels of NADH due to mutations in *ndh* (Miesel *et al*., 1998; Lee *et al*., 2001; Vilchèze *et al*., 2005); and titration of the INH-NAD adduct by increased expression of InhA due to promoter-up mutations (Larsen *et al*., 2002; Vilchèze *et al*., 2006). Importantly, resistance mechanisms for up to 22% of the INH-resistant *M. tuberculosis* clinical isolates still remain unknown (Hazbon *et al*., 2006).

The action of INH has been shown to be linked with NAD metabolism in *M. tuberculosis*, early studies showed that the NAD content of *M. tuberculosis* decreased when cells were exposed to INH (Bekierkunst, 1966). However, the NAD metabolism of *M. tuberculosis* has not been thoroughly investigated in spite of efforts made during the last few years (Boshoff *et al*., 2008; Vilchèze *et al*., 2010), and the link between INH and NAD metabolism is still unclear.

Ethionamide (ETH) is an important component of second-line therapy for the treatment of multidrug-resistant TB, and shares a common target with INH. Like INH, ETH is a pro-drug that requires activation to form adducts with NAD that subsequently inhibit InhA (Banerjee *et al*., 1994; Baulard *et al*., 2000; DeBarber *et al*., 2000; Vannelli *et al*., 2002; Wang *et al*., 2007). Resistance to ETH has been reported to result from various mechanisms, including mutations altering EthA/EthR (Baulard *et al*., 2000; DeBarber *et al*., 2000; Morlock *et al*., 2003), InhA and its promoter (Banerjee *et al*., 1994; Larsen *et al*., 2002; Vilchèze *et al*., 2006), the NADH dehydrogenase encoded by *ndh* (Miesel *et al*., 1998; Vilchèze *et al*., 2005), and the MshA enzyme which is involved in mycothiol biosynthesis (Vilchèze *et al*., 2008). However, the mechanism of resistance to ETH of 19% of the ETH-resistant isolates remains unknown (Brossier *et al*., 2011).

Rv3199c of *M. tuberculosis* H37Rv and BCG_3224c of *Mycobacterium bovis* BCG Pasteur strain 1173P2 (hereafter referred to as *M. bovis* BCG) encode a NADH pyrophosphatase (NudC, EC 3.6.1.22), which belongs to the Nudix hydrolase superfamily of nucleotide pyrophosphatases. The Nudix hydrolases comprise a large family of proteins characterized by a highly conserved 23-amino-acid Nudix motif (Nudix box), GX_5_EX_7_REUXEEXGU, where U represents a bulky, hydrophobic, amino acid, usually Ile, Leu or Val (Bessman *et al*., 1996). The sequence SQPWPFPQS located 10 residues downstream of the Nudix box is found in many characterized NADH hydrolases and may confer pyridine nucleotide specificity (Dunn *et al*., 1999). The Nudix superfamily proteins (InterPro IPR000086; PfamPF00293) act on substrates with a general structure (nucleoside diphosphate linked to another moiety X, NDP-X), yielding NMP and P-X (Bessman *et al*., 1996). The hydrolysis of dinucleotide pyrophosphates requires divalent metal ions (Mg^2+^ or Mn^2+^) and yields two mononucleoside 5′-phosphates (Frick and Bessman, 1995). In addition to NADH and NAD^+^, structurally related compounds like NADPH, ADP-ribose and diadenosine polyphosphates are also substrates of this superfamily of proteins (Frick and Bessman, 1995).

Since NudC has a rather broad range of substrates as mentioned above, we speculated that mycobacterial NudC can hydrolyse the INH-NAD and ETH-NAD adducts, resulting in the inactivation of these inhibitors. Thus, overexpression of NudC should lead to INH or ETH resistance in mycobacteria. On the contrary, inactivation of NudC should increase susceptibility to INH and ETH. To verify this hypothesis, we first characterized NudC from *M. tuberculosis* H37Rv (NudC_Rv_) and *M. bovis* BCG (NudC_BCG_) biochemically. Their abilities to hydrolyse INH-NAD or ETH-NAD were verified by mass-spectrometry and also enzymatic assays. The corresponding NudC-encoding genes were then overexpressed in *Mycobacterium smegmatis* mc^2^155 and *M. bovis* BCG to examine changes in drug susceptibility. Furthermore, *nudC* of *M. smegmatis* mc^2^155 and *M. bovis* BCG was deleted to look into the changes of drug susceptibilities.

## Results

### Alignment of NudC protein sequences from different origins

**Fig. 1:**
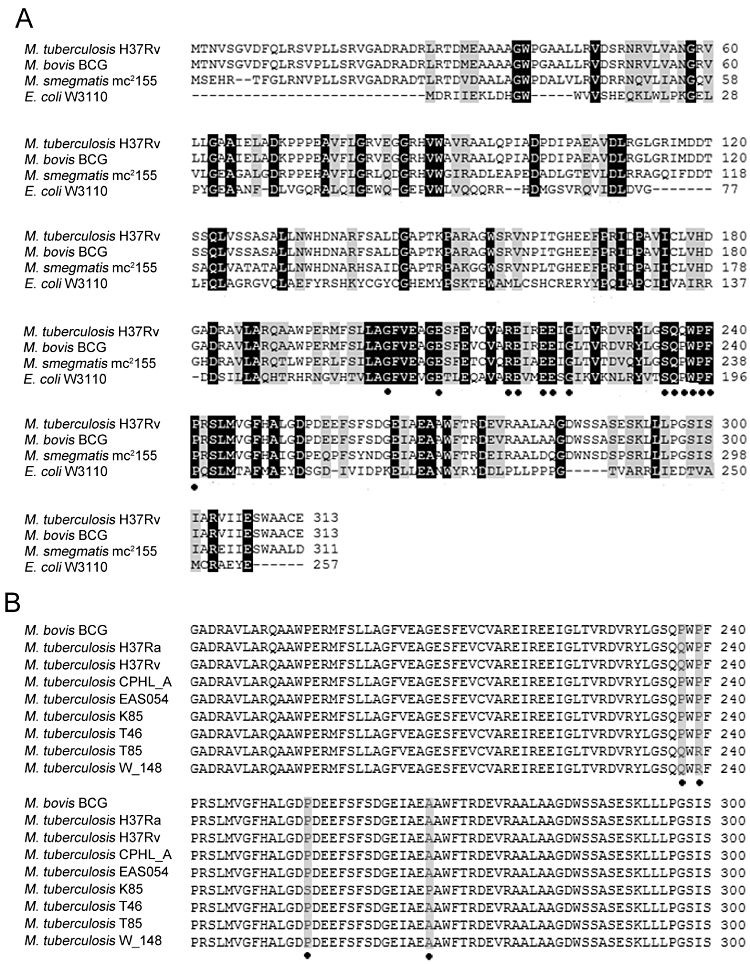
Multiple alignments of NudC from different origins. Sequences aligned using clustal w. A. Alignments of NudC from *E. coli* W3110 (AP_003822), *M. tuberculosis* H37Rv (NP_217715), *M. smegmatis* mc^2^155 (YP_886312) and *M. bovis* BCG (YP_979308). Conserved residues (Nudix box and NudC characteristic sequences) in NudC are highlighted (•). B. Alignments of NudC from different *M. tuberculosis* clinical isolates and *M. bovis* BCG. Only regions containing differences are highlighted (grey and •).

*Mycobacterium tuberculosis* H37Rv *nudC* gene (Rv3199c) encoding NudC consists of 942 base pairs corresponding to 313 amino acid residues. Multiple alignments of mycobacterial NudC show that *M. tuberculosis* NudC has a polymorphism (P237Q) not present in any other mycobacteria which lies in a very conserved region of NudC (SQPWPFPQS) and is predicted to confer pyridine nucleotide specificity (Dunn *et al*., 1999) (Fig. 1A). Subsequently, alignments of NudC between *M. bovis* BCG and *M. tuberculosis* clinical isolates (http://www.broadinstitute.org/annotation/genome/mycobacterium_tuberculosis_diversity/GenomesIndex.html) were also performed and multiple differences were noted (Fig. 1B). There were two amino acid differences (residues 254 and 268) between NudC from *M. bovis* BCG and *M. tuberculosis* K85 (Mostowy *et al*., 2004), and between *M. tuberculosis* T85 and *M. tuberculosis* w_148 (Hirsh *et al*., 2004; Gagneux *et al*., 2006) (residues 237 and 239). However, the sequence of NudC from the three *M. tuberculosis* clinical isolates *M. tuberculosis* T46, *M. tuberculosis* CPHL_A and *M. tuberculosis* EAS054 (Hirsh *et al*., 2004; Mostowy *et al*., 2004) was found to be identical to NudC_BCG_. *nudC* genes from a further 137 *M. tuberculosis* clinical isolates from China were sequenced and analysed (Table S1). Results showed that the P237Q mutation of NudC was present in all of these isolates. In addition, about 75% of these isolates harboured a second mutation (P239R) in the NudC conserved signature sequence, and a few isolates were found to harbour mutations at other sites.

### Physicochemical characterization of NudC_Rv_ and NudC_BCG_

**Fig. 2:**
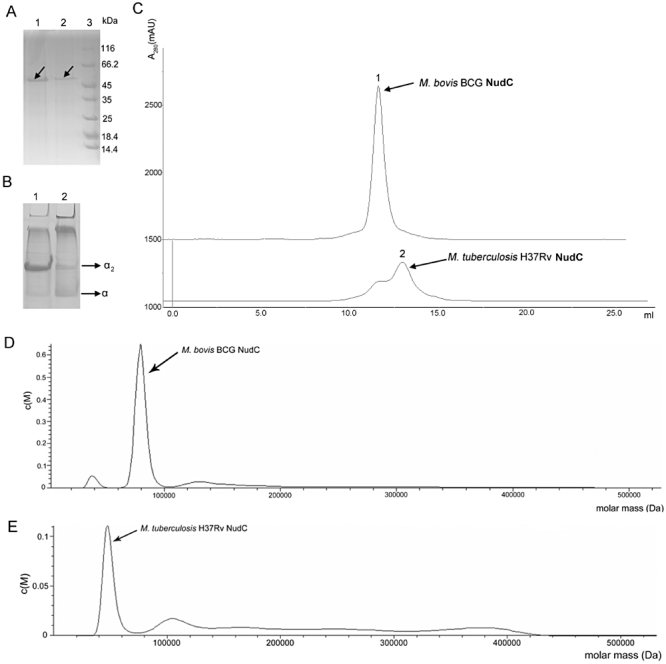
Analysis of NudC native structure. A. Nickel affinity-purified NudC_BCG_ (lane 1, arrow) and NudC_Rv_ (lane 2, arrow) were electrophoresed on 10% (v/v) SDS-PAGE; lane 3: protein molecular weight marker. B. Native PAGE of purified NudC_BCG_ (lane 1) and NudC_Rv_ (lane 2) on 9% (v/v) PAGE. Two NudC bands were detected and are indicated as α_2_ and α (arrows indicated). C. Gel exclusion chromatography analysis of the native structure of purified NudC_Rv_ and NudC_BCG_. Nickel affinity-purified NudC_BCG_ and NudC_Rv_ were concentrated then loaded onto a Superdex 200 10/300GL column and eluted. D and E. Analytical ultracentrifugation analysis of the native structure of purified NudC_BCG_ (D) and NudC_Rv_ (E). Expected products are indicated by arrows.

NudC_Rv_ and NudC_BCG_ were successfully expressed in *Escherichia coli* BL21 (DE3) by using the expression vector pET32a containing the thioredoxin protein (Trx) tag, which has been shown to be very effective in reducing inclusion body formation (LaVallie *et al*., 1993). SDS-PAGE analysis shows (Fig. 2A) that the molecular weight of purified recombinant NudC (fused with Trx tag) is about 51 kDa, which is consistent with its theoretical molecular weight.

The native molecular weight of recombinant NudC was determined by gel exclusion chromatography. As shown in Fig. 2C, NudC_Rv_ has two adjacent elution peaks (peak 1 and 2 as indicated by arrows) whereas NudC_BCG_ has only one peak (peak 1). Subsequent SDS-PAGE analysis revealed that both peaks 1 and 2 contained recombinant NudC proteins, NudC_BCG_ being present mainly in elution peak 1 while NudC_Rv_ was present mainly in peak 2 (Fig. 2C). According to results from elution curve analysis, it was clear that NudC_BCG_ was eluted faster than NudC_Rv_, suggesting that NudC_BCG_ and NudC_Rv_ may have two structural forms under native conditions. The native molecular weight of recombinant NudC was further analysed by analytical ultracentrifugation; recombinant NudC_BCG_ was estimated to be about 80 kDa, although a small portion of protein was estimated to be about 40 kDa (Fig. 2D). The molecular weight of NudC_Rv_ was estimated to be about 50 kDa, although a small portion of protein was estimated to be about 100 kDa (Fig. 2E). The molecular weights of recombinant NudC estimated from gel exclusion chromatography analysis were thus consistent with results from analytical ultracentrifugation. To further analyse differences in protein native structure between NudC_Rv_ and NudC_BCG_, non-denaturing PAGE experiments were performed (Fig. 2B). Under non-denaturing conditions two bands were observed for both proteins after staining. NudC_BCG_ was present mainly in the upper-band (possible dimeric form, indicated as α_2_), but NudC_Rv_ was present mainly in the lower band (possible monomeric form, indicated as α). These data, combined with data from gel exclusion chromatography and analytical ultracentrifugation, suggest that NudC_Rv_ is mainly present as a monomeric protein, while NudC_BCG_ is mainly dimeric under native conditions.

NudC in most *M. tuberculosis* clinical isolates from China harboured double mutations (P237Q and P239R) (hereafter referred to as NudC_QR_) which were identical to those of *M. tuberculosis* clinical isolates T85 and W_148. This NudC double mutant protein was purified and then analysed by gel exclusion chromatography (Fig. S1A) and native PAGE (Fig. S1B) as described above. Results showed that NudC_QR_ mainly forms a monomeric protein and small of dimeric protein under native conditions.

Taken together, our results indicate that residue 237 of NudC_Rv_ plays an important role in maintaining its dimeric structure. As a consequence, the mutation of this residue (P237Q) in *M. tuberculosis* NudC leads to the failure of protein dimer formation. In addition, residue 239 of NudC also affects dimer formation.

### Enzymatic assays of NudC_BCG_ and NudC_Rv_

Results from the alignment of *M. tuberculosis* NudC with NudC from other species show that the Nudix box and NudC characteristic sequences are highly conserved. NADH has already been shown to be the substrate favoured over all other nucleoside diphosphate derivatives by NudC from other species (Frick and Bessman, 1995; Bessman *et al*., 1996). In addition, divalent metal ions have been shown to be essential for NudC enzymic activity, with the most efficient stimulators being Mg^2+^ and Mn^2+^, as previously reported (Frick and Bessman, 1995).

NADH/NAD^+^ hydrolysis by NudC in the presence of different metal ions was verified according to a standard protocol (Frick and Bessman, 1995). Parallel experiments were performed for NudC_Rv_ and NudC_BCG_. As shown in Table 1, NudC_BCG_ and NudC_Rv_ differ in several respects. First, in the presence of Mg^2+^, NudC_Rv_ barely hydrolysed either NADH or NAD^+^, whereas hydrolysis by NudC_BCG_ was significant, and showed a marked preference for NADH over NAD^+^. Second, in the presence of Mn^2+^, NudC from both species could hydrolyse NADH, but the specific activity of NudC_Rv_ was only approximately one-fifth that of NudC_BCG_. Third, even in the presence of Mn^2+^, NudC_Rv_ could barely hydrolyse NAD^+^. Other than Mg^2+^ and Mn^2+^, NudC_BCG_ showed some activity on NADH in the presence of Fe^2+^ as well. However, neither enzyme had detectable activity in the presence of Ca^2+^, Cu^2+^, Zn^2+^ or Fe^3+^.

**Table 1:** Comparison of specific activities of NudC_Rv_ and NudC_BCG_ in the presence of different metal ions^a^

	*M. bovis* BCG (U·mg^−1^)		*M. tuberculosis* H37Rv (U·mg^−1^)	
		
	NADH	NAD^+^	NADH	NAD^+^
Mg^2+^	1.59 ± 0.2	0.03 ± 0.01	0.02 ± 0.01	0
Mn^2+^	1.44 ± 0.1	0.58 ± 0.08	0.29 ± 0.1	0.05 ± 0.03
Zn^2+^	0.06 ± 0.01	0.01 ± 0.01	0.05 ± 0.01	0.04 ± 0.01
Ca^2+^	0	0	0	0
Cu^2+^	0	0	0	0
Fe^3+^	0.02 ± 0.01	0	0.03 ± 0.01	0
Fe^2+^	0.39 ± 0.05	0.05 ± 0.02	0.03 ± 0.01	0

a Measurements were performed in triplicate. Values are means ± standard deviations.

In addition to NADH and NAD^+^, several nucleotide pyrophosphate analogues (NADP^+^, NADPH, ADP-ribose) were also tested as substrates for NudC_Rv_ and NudC_BCG_, and their relative activities are shown in Table S2. As expected, NADH was favoured over all other substrates tested. Both enzymes preferred the reduced forms of the substrates, consistent with results for NudC from other sources (Xu *et al*., 2000). NudC_BCG_ was found to have a broader range of substrates than NudC_Rv_.

The enzymatic activity of NudC_QR_ from a representative *M. tuberculosis* clinical isolate was also determined. The enzymatic activity of this double mutant could hardly be detected in the presence of any metal ion (Mg^2+^, Mn^2+^ or any other ion), no matter what substrate was used.

Taken together, these results indicate that residue 237 of *M. tuberculosis* NudC is important, not only for its dimerization, but also for its metal ion binding and enzymatic activity. Residue 239 may also play a role in its enzymatic activity.

Using NADH as the substrate, the optimal pH and temperature of NudC_BCG_ were determined to be pH 8.0 and 40°C respectively (Fig. S2A and B). However, enzyme activity rapidly decreased below pH 7.5, or when the temperature was below 35°C or above 45°C.

### NudC_BCG_ hydrolyses the INH-NAD and ETH-NAD adducts

As described above, NudC from both *M. bovis* BCG and *M. tuberculosis* were verified to be NADH pyrophosphatases, and NudC_BCG_ was shown to have a rather broad range of substrates. Since INH-NAD and ETH-NAD adducts are analogues of NAD, we wondered whether NudC_BCG_ might be also able to hydrolyse it. To test this hypothesis, INH-NAD and ETH-NAD adducts were synthesized and purified according to published protocols (Lei *et al*., 2000; Nguyen *et al*., 2001; 2002; Wang *et al*., 2007). Synthesized INH-NAD (Fig. S3) and ETH-NAD (Fig. S4) adducts were analysed by HPLC-MS (Figs S3A and S4A) and UV/VIS spectrometry (Figs S3B and S4B), and results obtained were found to be consistent with previous reports (Lei *et al*., 2000; Nguyen *et al*., 2001; 2002; Wang *et al*., 2007).

**Fig. 3:**
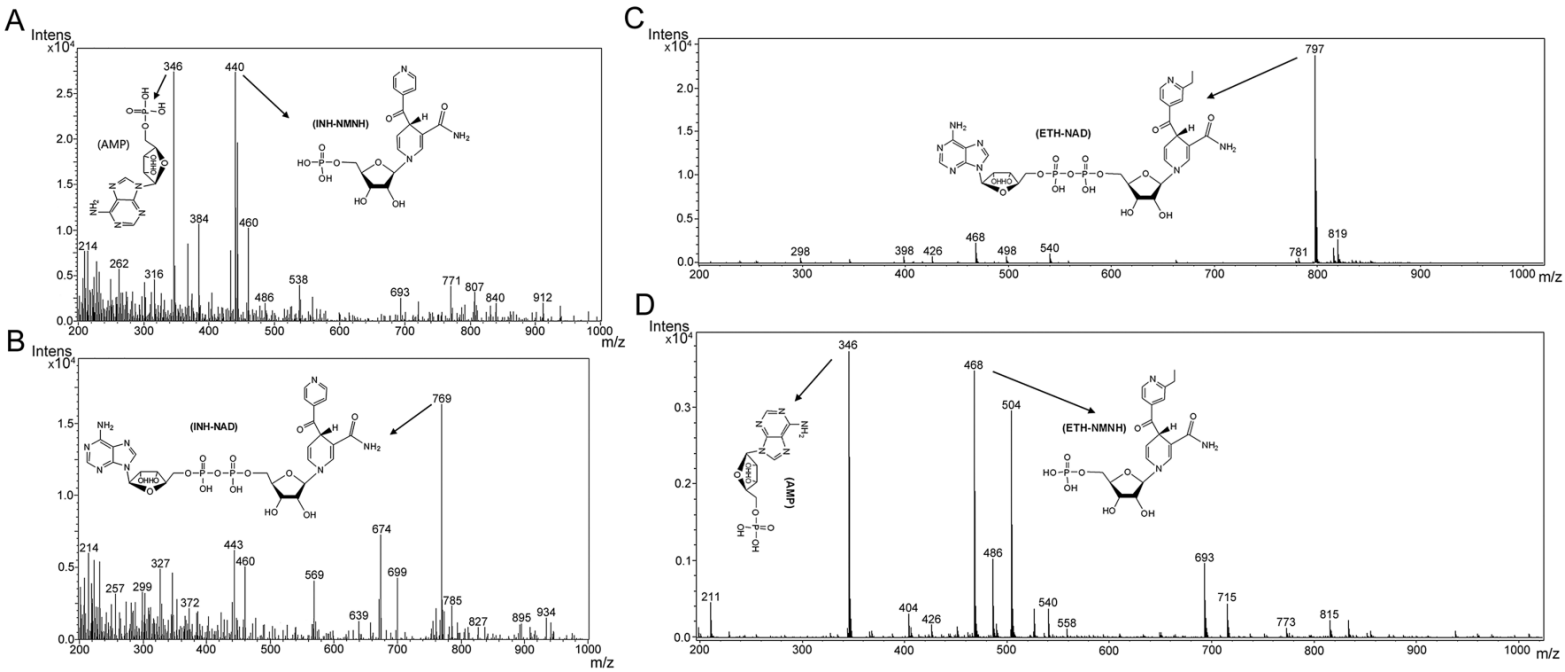
HPLC-MS analysis of the INH-NAD and ETH-NAD adducts hydrolysed by NudC. Purified NudC was incubated with INH-NAD (A and B) or ETH-NAD (C and D) adducts in the presence of Mg^2+^, reacted sufficiently, and the reaction mixture was then filtered to remove protein before analysing the filtrate by HPLC-MS. The data from mass spectral analysis of the NudC_BCG_ hydrolysis products are shown in (A) and (D), and those of NudC_Rv_ are shown in (B) and (C). The product peaks are indicated by arrows and chemical structures showing the composition and structure of the molecules are shown. INH-NAD, calculated weight = 770 and found weight = 769 [(M-H)^-^]; ETH-NAD, calculated weight = 798 and found weight = 797 [(M-H)^-^]; AMP, calculated weight = 347 and found weight = 346 [(M-H)^-^]; INH-NMNH, calculated weight = 441 and found weight = 440 [(M-H)^-^]; ETH-NMNH, calculated weight = 469 and found weight = 468 [(M-H)^-^]. All MS data were acquired in the negative mode.

After incubation of INH-NAD or ETH-NAD with NudC, the reaction mixture was filtered through a Microcon 3 centricon filter unit to remove all proteins. Filtrates collected were then analysed by HPLC-MS, and results indicated that NudC_BCG_ could hydrolyse the INH-NAD and ETH-NAD adducts in the presence of Mg^2+^, yielding AMP [(M-H)^-^] (peak of m/z 346) and INH-NMNH [(M-H)^-^] (peak of m/z 440) (Fig. 3A) or AMP [(M-H)^-^] (peak of m/z 346) and ETH-NMNH [(M-H)^-^] (peak of m/z 468) (Fig. 3D). However, NudC_Rv_ could not hydrolyse the INH-NAD or ETH-NAD adducts, and only had a peak of m/z 769 [(M-H)^-^] representing the intact INH-NAD adduct (Fig. 3B) or a peak of m/z 797 [(M-H)^-^] representing the ETH-NAD adduct (Fig. 3C).

Results indicated that the NudC_QR_ could not hydrolyse INH-NAD or ETH-NAD adduct, and only had a peak of m/z 769 [(M-H)^-^] representing the intact INH-NAD adduct (Fig. S5) or m/z 797 [(M-H)^-^] representing the ETH-NAD adduct (Fig. S6).

### Overexpression of NudC in mycobacteria

The *nudC* gene from *M. bovis* BCG and *M. tuberculosis* H37Rv were both cloned into pMV261. The resulting plasmids pMV261-NudC_BCG_, pMV261-NudC_Rv_ and empty vector pMV261 were electroporated into *M. smegmatis* mc^2^155, yielding mc^2^155 pMV261::*nudC*_BCG_, mc^2^155 pMV261::*nudC*_Rv_ and mc^2^155 pMV261 respectively. To confirm the overexpression of NudC_Rv_ and NudC_BCG_*in vivo*, polyclonal anti-NudC_Rv_ and anti-NudC_BCG_ antibodies were raised. Western blotting showed that NudC from both species was expressed successfully in *M. smegmatis* mc^2^155 (Fig. S7).

As mentioned above, since NudC_BCG_ is able to hydrolyse the INH-NAD and ETH-NAD adducts and thus inactivate them, overexpression of this gene should result in INH and ETH resistance. Subsequently, tests for drug susceptibility to INH, ETH, Rifampicin (RIF) and Ethambutol (EMB) were performed on the NudC_BCG_ overexpressing *M. smegmatis* mc^2^155 strain (mc^2^155 pMV261::*nudC*_BCG_). As shown in Table 2, mc^2^155 pMV261::*nudC*_BCG_ was co-resistant to INH and ETH, with 20- to 40-fold increases in MIC (Minimum inhibitory concentration) for each compound, but its susceptibility to the other two drugs was not changed. The susceptibility of mc^2^155 pMV261::*nudC*_Rv_ and mc^2^155 pMV261 to these four drugs was not changed. These results suggest that the co-resistance of mc^2^155 pMV261::*nudC*_BCG_ to INH and ETH was caused by overexpression of NudC_BCG_, and not from other causes. Similarly, overexpression of *M. smegmatis* mc^2^155 NudC (NudC_Sm_) in *M. smegmatis* mc^2^155 (mc^2^155 pMV261::*nudC*_Sm_) also resulted in a high level of resistance to both INH and ETH and susceptibility to the other two drugs (RIF and EMB) was not changed (Table 2).

**Table 2:** Drug susceptibility tests of mycobacterial strains

		MIC (µg ml^−1^)			
		
Strain	Description	INH	ETH	EMB	RIF
mc^2^155	*M. smegmatis* mc^2^155	5	10	0.5	10
mc^2^155 pMV261	mc^2^155 transformed with pMV261	5	10	0.5	10
mc^2^155 pMV261::*nudC*_Rv_	mc^2^155 transformed with pMV261-NudC_Rv_	5	10	0.5	10
mc^2^155 pMV261::*nudC*_BCG_	mc^2^155 transformed with pMV261-NudC_BCG_	> 200	200	0.5	10
mc^2^155 Δ*nudC*	mc^2^155 knockout *nudC*	1	0.5	0.5	10
mc^2^155 pMV261::*nudC*_Sm_	mc^2^155 transformed with pMV261-NudC_Sm_	200	50	0.5	10
BCG	*M. bovis* BCG Pasteur1173P2	0.1	5	2.5	0.1
BCG pMV261	BCG transformed with pMV261	0.1	5	2.5	0.1
BCG pMV261::*nudC*_Rv_	BCG transformed with pMV261-NudC_Rv_	0.1	5	2.5	0.1
BCG pMV261::*nudC*_BCG_	BCG transformed with pMV261-NudC_BCG_	> 2	> 50	2.5	0.1
BCG Δ*nudC*	BCG knockout *nudC*	0.05	1	2.5	0.1
H37Ra	*M. tuberculosis* H37Ra	0.1	1	2.5	0.01
H37Ra Δ*nudC*	H37Ra knockout *nudC*	0.1	1	2.5	0.01
mc^2^155 Δ*nudC* pMV261::*nudC*_Sm_	mc^2^155 Δ*nudC* transformed with pMV261-NudC_Sm_	> 200	200	0.5	10
BCG Δ*nudC* pMV261::*nudC*_BCG_	BCG Δ*nudC* transformed with pMV261-NudC_BCG_	> 2	> 50	2.5	0.1

Recombinant plasmids pMV261-NudC_BCG_, pMV261-NudC_Rv_ and empty vector pMV261 were electroporated into *M. bovis* BCG yielding BCG pMV261::*nudC*_BCG_, BCG pMV261::*nudC*_Rv_ and BCG pMV261 respectively. We found that the efficiency of transformation of pMV261-NudC_BCG_ was extremely low in comparison with that of pMV261-NudC_Rv_ and the blank vector pMV261 (less than 20 transformants per µg plasmid versus thousands of transformants per µg plasmid). To verify the transformants obtained, specific sequences from the overexpression vector containing *nudC* were amplified and then sequenced. Interestingly, most sequences amplified from pMV261-NudC_BCG_ transformants contained deletions and mutations of the *nudC* gene, but there were no mutations in the chromosomal copy of *nudC* gene (data not shown), and only a very few transformants (about one in 30) had an intact coding sequence of NudC, indicating that toleration of the overexpression of NudC may require secondary mutations. A very similar phenomenon was also observed in *M. tuberculosis* H37Ra, suggesting that unlike *M. smegmatis* mc^2^155, overexpression of functional NudC in slow-growing mycobacteria is not tolerated. This may reflect differences in NAD metabolism between fast and slow growing mycobacteria.

Subsequent drug susceptibility tests showed that, BCG pMV261::*nudC*_BCG_ was also co-resistant to INH and ETH, whereas the susceptibility of BCG pMV261::*nudC*_Rv_ and BCG pMV261 to these two drugs was unchanged (Table 2). Susceptibility to RIF and EMB in these strains also remained unchanged (Table 2).

### Inactivation of *nudC* resulted in increased susceptibility to both INH and ETH

To further investigate the physiological role of mycobacterial *nudC* on cellular metabolism of INH and ETH, the *nudC* gene was deleted from *M. smegmatis* mc^2^155, *M. bovis* BCG and *M. tuberculosis* H37Ra and verified by PCR (Fig. S8). As expected, the *M. smegmatis* mc^2^155, *M. tuberculosis* H37Ra and *M. bovis* BCG strains in which *nudC* was knocked out, mc^2^155 Δ*nudC* and BCG Δ*nudC*, were more sensitive to both INH and ETH in MIC testing; however, the susceptibility of the *M. tuberculosis* H37Ra *nudC* knock out strain (H37Ra Δ*nudC*) to these two drugs was unchanged (Table 2). Complemented strains mc^2^155 Δ*nudC* pMV261::*nudC*_Sm_ and BCG Δ*nudC* pMV261::*nudC*_BCG_ also showed resistance to both INH and ETH (Table 2), consistent with results obtained in our *nudC* overexpression experiments. NudC was also overexpressed in the complemented strains and the level of NudC expressed was much higher than that in the wild-type strain, also conferring resistance to INH and ETH on these complemented strains. Susceptibilities to RIF and EMB remained unchanged in these strains (Table 2).

**Fig. 4:**
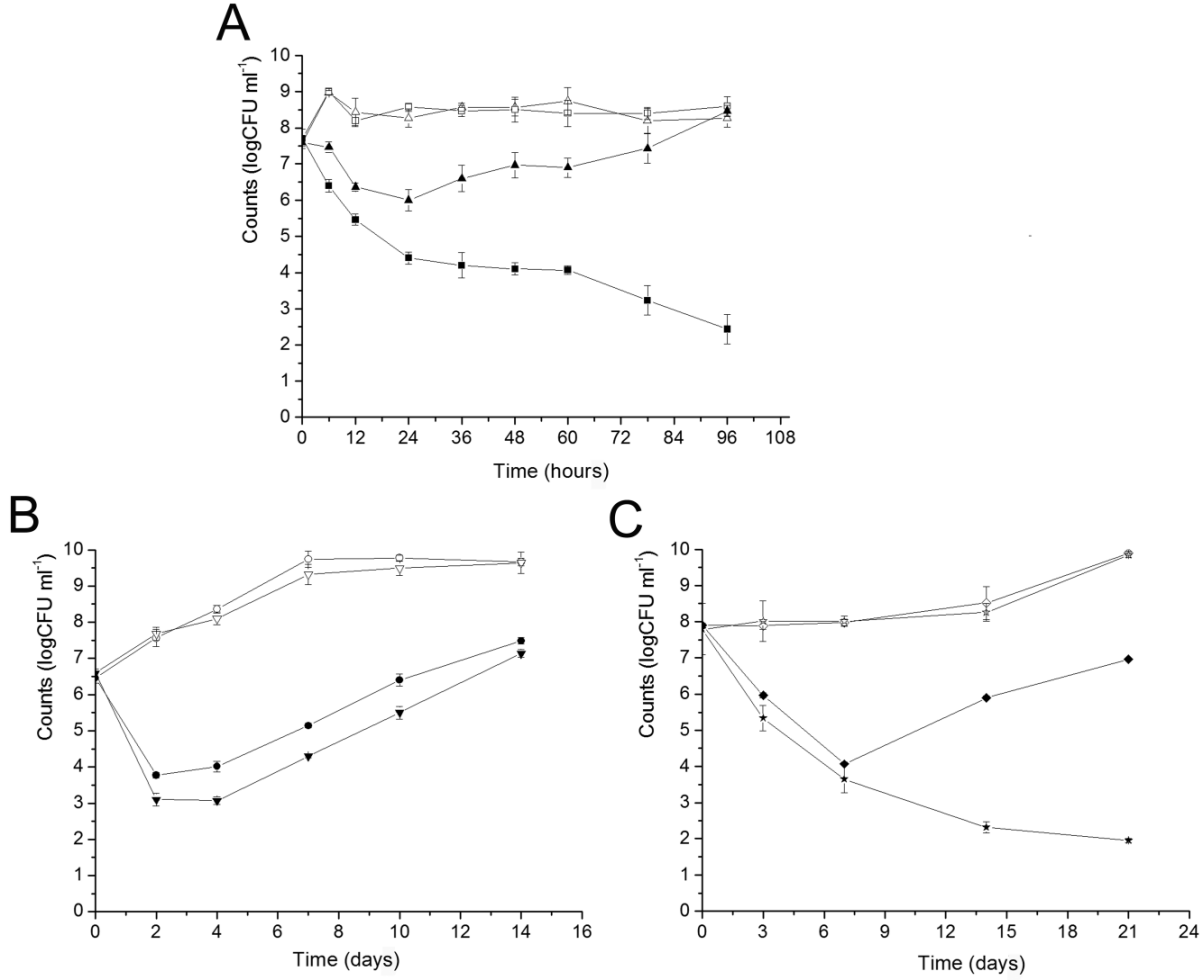
INH and ETH mycobacterial killing curves. Mycobacterial cells were grown to mid-log phase (OD_600_ of 0.5–1.0) and diluted to about 10^6^–10^7^ cfu ml^−1^ in fresh medium. INH or ETH was then added and aliquots were removed at regular intervals. Serial dilutions were performed before plating. A. *M. smegmatis* mc^2^155 (▵) and mc^2^155 Δ*nudC* (□) with no drug added; *M. smegmatis* mc^2^155 (▴) and mc^2^155 Δ*nudC* (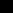) with 50 µg ml^−1^ INH added. B. *M. bovis* BCG (○) and BCG Δ*nudC* (▿) with no drug added; *M. bovis* BCG (•) and BCG Δ*nudC* (▾) with 5 µg ml^−1^ INH added. C. *M. bovis* BCG (◊) and BCG Δ*nudC* (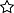) with no drug added; *M. bovis* BCG (⋄) and BCG Δ*nudC* (★) with 50 µg ml^−1^ ETH added. Experiments were performed in triplicate. Standard deviations are indicated by error bars.

Drug exposure experiments were performed and results showed that both mc^2^155 Δ*nudC* and BCG Δ*nudC* had a defect in the INH tolerance phenotype (Fig. 4A and B). The INH killing curve of mc^2^155 Δ*nudC* differed from that of the wild-type strain. Deletion of *nudC* resulted in greater killing in the mc^2^155 Δ*nudC* mutant strain throughout the course of treatment (96 h) than in the wild-type strain at the same concentration of INH. In the wild-type strain, the killing effect of INH peaked after 24 h of treatment, and then bacteria started to grow again. In the mc^2^155 Δ*nudC* mutant, the killing effect of INH was plateaued between 24 and 60 h, but increased thereafter as INH continued to inhibit the growth of the bacteria (Fig. 4A).

Although deletion of *M. bovis* BCG *nudC* only resulted in an about 1 log-fold increase in killing by INH compared with the wild-type strain (Fig. 4B), it led to continuous killing by ETH (Fig. 4C). In the wild-type strain, the killing effect of ETH peaked after 7 days of treatment, and then bacteria began to grow again. However, in the BCG Δ*nudC* mutant, the killing effect continued to 21 days of treatment.

These results provide further strong support showing that, although previously unrecognized, mycobacterial NudC plays a role in the cellular metabolism of INH and ETH which is very important for the action and also the tolerance of these two drugs.

### Mycolic acid analysis of mycobacteria following INH or ETH treatment

**Fig. 5:**
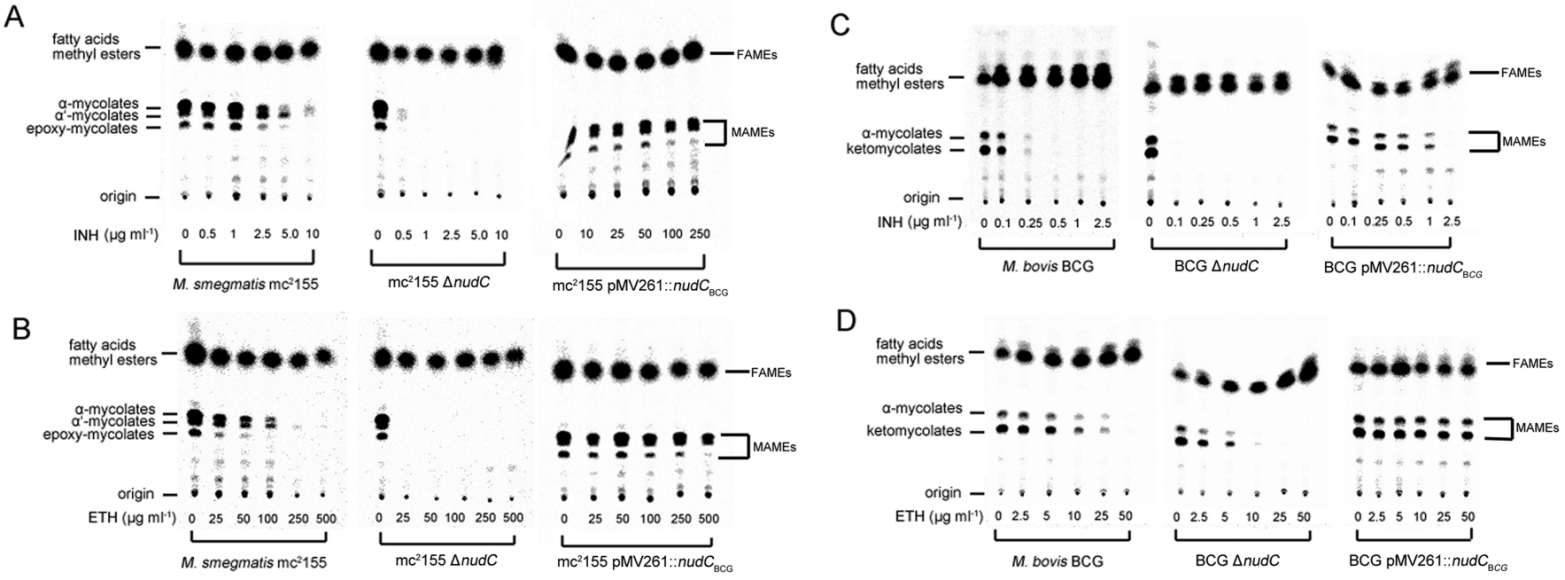
Inhibition of mycobacterial mycolic acid biosynthesis by INH and ETH. *M. smegmatis* mc^2^155, mc^2^155 Δ*nudC* and mc^2^155 pMV261::*nudC*_BCG_ were treated with serial concentrations of INH (A) or ETH (B) for 4 h, and then labelled with 1, 2-[^14^C] acetate for another 4 h. FAMEs and MAMEs were then extracted and separated by TLC. ^14^C-labelled FAMEs and MAMEs were detected by autoradiography after overnight exposure to a Kodak BioMax MR film. Similarly, *M. bovis* BCG, BCG Δ*nudC* and BCG pMV261::*nudC*_BCG_ were treated with serial concentrations of INH (C) or ETH (D) for 24 h. Then 1, 2-[^14^C] acetate was added and cultures were further incubated for another 24 h. FAMEs and MAMEs were then extracted and separated by TLC. ^14^C-labelled FAMEs and MAMEs were detected by autoradiography after overnight exposure to a Kodak BioMax MR film.

As mentioned above, overexpression of NudC_BCG_ in *M. smegmatis* mc^2^155 and *M. bovis* BCG resulted in resistance to INH and ETH, while deletion of the *nudC* gene in *M. smegmatis* mc^2^155 and *M. bovis* BCG caused increased susceptibility to both drugs. Since treatment with INH or ETH leads to inhibition of mycobacterial mycolic acid biosynthesis, we expect that changes in susceptibility to these drugs resulting from overexpression of NudC or deletion of the *nudC* gene may alter their effect on mycobacterial mycolic acid biosynthesis. Mycobacterial strains (*M. smegmatis* mc^2^155, mc^2^155 Δ*nudC*, mc^2^155 pMV261::*nudC*_BCG_, *M. bovis* BCG, BCG Δ*nudC* and BCG pMV261::*nudC*_BCG_) were grown in the presence INH and ETH at various concentrations, after which cultures were labelled with 1, 2-[^14^C] acetate. Combined fatty acid methyl esters (FAMEs) and mycolic acid methyl esters (MAMEs) were extracted, resolved and fractionated on TLC plates. Treatment of mc^2^155 Δ*nudC* with INH (from 0 to 10 µg ml^−1^) (Fig. 5A) or ETH (from 0 to 500 µg ml^−1^) (Fig. 5B) resulted in very strong inhibition of mycolic acid biosynthesis as shown by the absences of MAMEs at a relatively low concentration of each drug (1 µg ml^−1^ INH, 25 µg ml^−1^ ETH) compared with the wild-type strain. On the contrary, INH (from 0 to 250 µg ml^−1^) and ETH (from 0 to 500 µg ml^−1^) showed very weak or nearly no inhibition of mycolic acid biosynthesis in NudC_BCG_ overexpressed *M. smegmatis* mc^2^155 strain (mc^2^155 pMV261::*nudC*_BCG_) (Fig. 5A and B). Similarly, INH and ETH inhibition of mycolic acid biosynthesis in BCG Δ*nudC* was markedly stronger compared with that in the wild-type strain, while it was drastically diminished by overexpression of NudC_BCG_ (Fig. 5C and D). INH and ETH at relatively low concentrations (0.1 µg ml^−1^ INH, 10 µg ml^−1^ ETH) completely abolished MAMEs synthesis in BCG Δ*nudC*, while higher concentrations of the drugs (0.5 µg ml^−1^ INH, 50 µg ml^−1^ ETH) were required to achieve the same effect in the wild-type strain. On the other hand, treatment of the NudC_BCG_ overexpressing strain BCG pMV261::*nudC*_BCG_ with INH (from 0 to 1 µg ml^−1^) or ETH (from 0 to 50 µg ml^−1^) had a rather weak or nearly no effect on the synthesis of MAMEs and FAMEs.

## Discussion

In this study, we report a unique character of *M. tuberculosis* NAD metabolism, and propose a novel mechanism of INH and ETH inactivation in mycobacteria.

NudC, the NADH pyrophosphatase, is a component of NAD salvage pathway in many bacteria species including mycobacteria. Protein sequence alignment showed that NudC from *M. tuberculosis* H37Rv harbours a polymorphism at residue 237 that is not present in other mycobacteria. Although this residue is conserved among NudC from different species and is also predicted to be one of the active site of the enzyme, so far no experimental data have been presented for this specific residue. In this study, through comparative biochemical analysis of purified NudC_BCG_ and NudC_Rv_, we found that the mutation at residue 237 results in disruption of protein dimer formation, changes in metal ion binding and loss of enzymatic activity. In addition, sequence analysis of *nudC* genes amplified from 137 *M. tuberculosis* clinical isolates from China revealed that *nudC* from most isolates harboured another mutation (residue 239), and a few isolates even had multiple mutations. The standard laboratory strain *M. tuberculosis* H37Rv was isolated in the year 1905. Since it was not possible to analyse the sequence of *nudC* from different clinical isolates at that time, it is now unclear whether the additional mutations of *nudC* identified in clinical isolates here were already present then or whether they have been acquired subsequently. Biochemical analysis of the purified double mutant NudC_QR_ showed that the mutations at residues 237 and 239 lead to complete loss of enzymatic activity. Alignment of mycobacterial NudC and *E. coli* NudC revealed that residues P237 and P239 are both located in a very conserved region (SQPWPFPQS) predicted to confer pyridine nucleotide specificity (Dunn *et al*., 1999) (Fig. 1B), suggesting that the mutation of these two residues may affect the binding of substrates. According to the results of amino acid sequence alignment, residue 237 of NudC_Rv_ corresponds to residue 193 of *E. coli* NudC. The crystal structure of *E. coli* NudC (PDB code 1vk6 and PDB code 2gb5) has been resolved and it was found to be a dimeric protein. Residue 193 of *E. coli* NudC is located on the surface of the interface between subunits (Fig. S9A and B). It may thus be the case that the mutation of residue 237 in NudC_Rv_ affects the interaction between subunits, causing the failure in protein dimerization observed here. In addition, residue 239 of NudC_Rv_ corresponds to residue 195 in *E. coli* NudC. Residue 195 is also known to be close to the surface of the protein interface between subunits (Fig. S9A and B). Thus it is not surprising to find that double mutation of residues 237 and 239 in NudC_QR_ also affect protein dimer formation. However, it is still remains unclear whether protein dimerization is necessary for the enzymatic activity of NudC.

From the above data, it is very clear that, in contrast to *M. bovis* BCG and other mycobacteria, *M. tuberculosi*s H37Rv and most *M. tuberculosi*s clinical isolates have acquired mutations in the *nudC* gene, leading to the loss of NADH pyrophosphatase activity. As mentioned above, both residues 237 and 239 are located in a conserved region (SQPWPFPQS) of NudC that is predicted to confer pyridine nucleotide specificity. It is possible that NudC_Rv_ may have gained additional as yet unknown functions, which remain to be identified in further studies.

One unique property of *M. tuberculosis* NAD metabolism is that it secretes niacin into the growth medium under *in vitro* growth conditions, and previous research revealed that, niacin secretion of *M. tuberculosis* was mainly due to its high NAD glycohydrolase activity (Kasărov and Moat, 1972). Here we report another unique property of *M. tuberculosis* NAD metabolism. Most *M. tuberculosis* clinical isolates have an inactive NADH pyrophosphatase, although the reason remains unknown.

Previous studies have shown that NudC has a rather broad range of substrates, including NADH/NAD^+^ and nucleoside diphosphate derivatives (Frick and Bessman, 1995). This raises the possibility that, as homologues of NAD, INH-NAD and ETH-NAD adducts could also be substrates of NudC. We validated this hypothesis here by biochemical assays including HPLC-MS. As expected, NudC_BCG_ was able to hydrolyse both adducts whereas NudC_Rv_ was not. Since NudC_BCG_ was capable of hydrolysing INH-NAD and ETH-NAD, we reasoned that its overexpression should lead to co-resistance to both drugs, and that deletion of the *nudC* gene should result in increased sensitivity to both drugs. This speculation was verified by *in vivo* experiments. Overexpression of NudC_BCG_ in *M. smegmatis* mc^2^155 and *M. bovis* BCG resulted in co-resistance to INH and ETH, but overexpression of NudC_Rv_ had no effect on susceptibility to both drugs. Meanwhile, overexpression of NudC_Sm_ in *M. smegmatis* mc^2^155 also resulted in co-resistance to INH and ETH, suggesting that NudC_Sm_ is also functional and able to hydrolyse INH-NAD and ETH-NAD adducts. On the other hand, deletion of the *nudC* gene in *M. smegmatis* mc^2^155 and *M. bovis* BCG resulted in increased susceptibility to INH and ETH in MIC tests. More interestingly, inactivation of the *nudC* gene also caused a defect in the INH or ETH tolerance phenotype (Fig. 4). Furthermore, data from the analysis of mycolic acids showed that overexpression of a functional NudC effectively alleviated the inhibition of mycolic acid biosynthesis caused by INH and ETH, and deletion of *nudC* elevated the effects of both drugs on mycolic acid biosynthesis (Fig. 5).

**Fig. 6:**
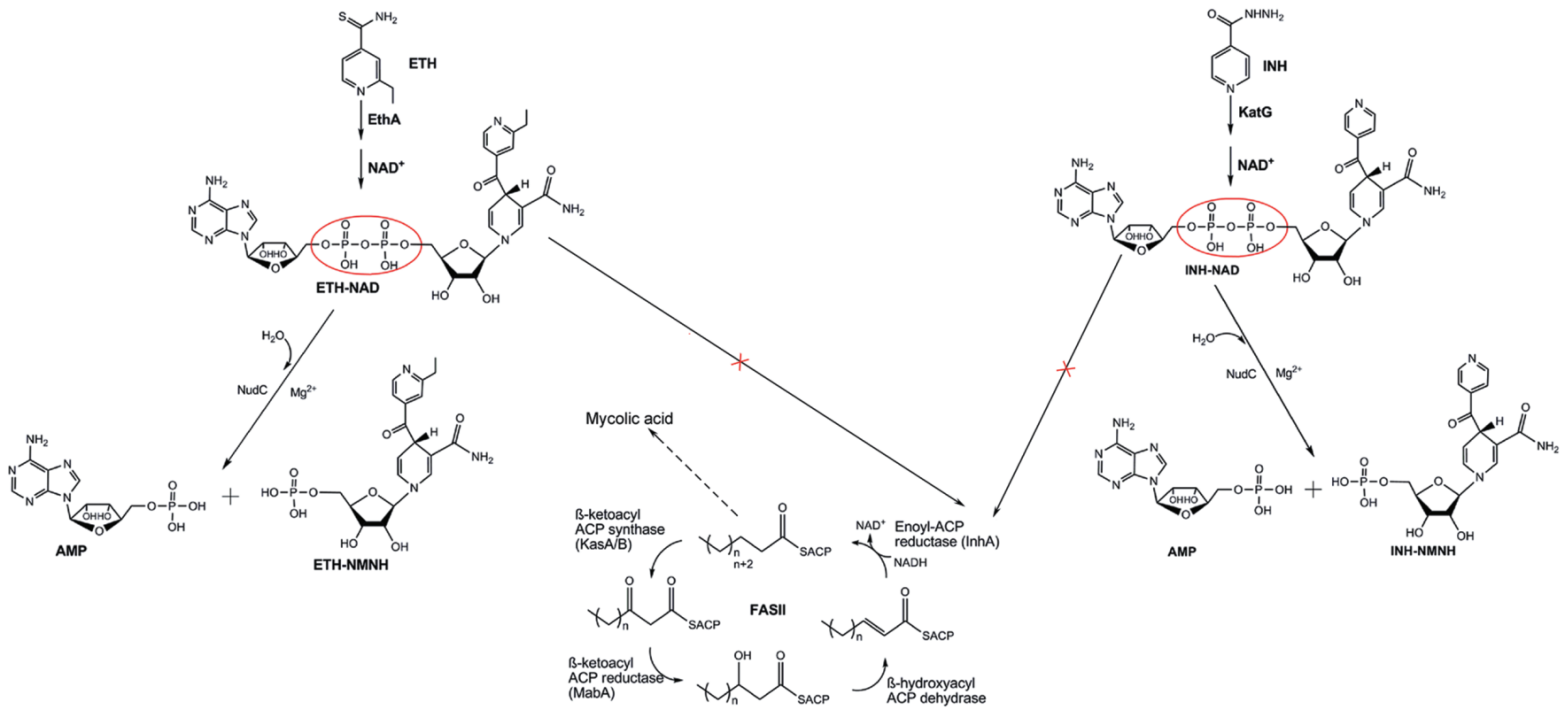
Diagram of a novel mechanism of INH an ETH inactivation. The INH prodrug requires activation by catalase-peroxidase KatG or Mn^3+^. Similarly, the ETH prodrug is activated by the flavin monooxygenase EthA. Activated intermediate products then react with NAD^+^ and yield an INH-NAD or ETH-NAD adduct. These adducts inhibit target protein InhA, the NADH-dependent enoyl-ACP reductase of the fatty acid synthase type II system, resulting in inhibition of mycolic acid biosynthesis and cell lysis. However, functional NudC hydrolyses the INH-NAD and ETH-NAD adducts, resulting in inactivation of these adducts and INH or ETH inactivation.

Based on the above results, we propose a novel mechanism of INH and ETH inactivation in mycobacteria, as shown in Fig. 6: INH and ETH are activated by KatG or EthA, then form INH-NAD and ETH-NAD adducts with NAD which inhibit the activity of InhA and result in inhibition of mycolic acid biosynthesis. Functional mycobacterial NudC, however, can hydrolyse INH-NAD and ETH-NAD adducts, blocking the effects of these drugs.

Inactivation of ETH and INH by mycobacterial NudC was first discovered in this manuscript; however, inactivation of INH by another mycobacterial enzyme Arylamine *N*-acetyltransferase (NAT) was observed by other researchers previously (Upton *et al*., 2001). The new finding that mycobacterial NudC can also inactivate INH adds to the complexity of metabolism of this drug in mycobacteria.

Except for *M. bovis* BCG, which was verified to be able to inactivate INH and ETH through the new mechanism, *M. smegmatis* mc^2^155 may also be able to inactivate both drugs by the same mechanism according to our results. Therefore it is important to investigate whether this new mechanism exists in other mycobacteria, especially other members of the *M. tuberculosis* complex. When comparing the NudC sequence of *M. bovis* BCG with those from clinical isolates of *M. tuberculosis* with known genome sequences (http://www.broadinstitute.org/annotation/genome/mycobacterium_tuberculosis_diversity/GenomesIndex.html), identical NudC sequences were found in the three *M. tuberculosis* clinical isolates T46, CPHL_A and EAS054 (Hirsh *et al*., 2004; Mostowy *et al*., 2004). This indicates that, although the NudC from *M. tuberculosis* H37Rv and most *M. tuberculosis* clinical isolates is inactive, intact and functional NudC exists in a small portion of *M. tuberculosis* clinical isolates, implying the new mechanism of INH and ETH inactivation may also exist in these isolates. This needs to be verified by further studies.

## Experimental procedures

### Bacterial strains, plasmids and media

Strains used are listed in Table S3. *E. coli* HB101 and *E. coli* TOP10 were used for constructing mycobacterial mutant strains. *E. coli* BL21 (DE3) was used as a host strain for recombinant protein expression. *M. smegmatis* mc^2^155, *M. bovis* BCG and *M. tuberculosis* H37Ra were kindly provided by Dr William R. Jacobs Jr (Howard Hughes Medical Institute, Albert Einstein College of Medicine). Plasmids pET32a, pET28a (Novagen) and pMV261 (kindly provided by Dr Guofeng Zhu) were used for construction of expression plasmids. *M. smegmatis* mc^2^155 was grown in Middlebrook 7H9 Broth or 7H10 agar medium (Difco) supplemented with 0.5% (v/v) glycerol and 0.05% (v/v) Tween-80. *M. bovis* BCG and *M. tuberculosis* H37Ra were grown in Middlebrook 7H9 Broth or 7H10 agar medium (Difco) supplemented with 10% (v/v) OADC (Difco), 0.5% (v/v) glycerol and 0.05% (v/v) Tween-80. Antibiotics (Sigma) were added at the following concentrations: kanamycin, 25 µg ml^−1^ for mycobacteria and 50 µg ml^−1^ for *E. coli*; hygromycin, 75 µg ml^−1^ for mycobacteria and 150 µg ml^−1^ for *E. coli*, and ampicillin, 100 µg ml^−1^ for *E. coli*.

### Molecular cloning

To generate expression constructs for purification of NudC, the *nudC* gene was amplified from *M. bovis* BCG and *M. tuberculosis* H37Rv genomic DNA using primers NudCNcoIFP and NudCXhoIRP (Table S3), digested with NcoI and XhoI, and then ligated to the expression vector pET32a, yielding pET32a-NudC_BCG_ and pET32a-NudC_Rv_. To generate *in vivo* overexpression constructs, *nudC* from *M. tuberculosis* H37Rv and *M. bovis* BCG were amplified using primers 261NudCBamHIFP and 261NudCHindIIIRP (Table S3), digested with BamHI and HindIII, and then cloned into pMV261, yielding pMV261-NudC_BCG_ and pMV261-NudC_Rv_. *nudC* from *M. smegmatis* mc^2^155 was amplified using primers 261NudCBamHIFP_Sm_ and 261NudCHindIIIRP_Sm_ (Table S3), digested with BamHI and HindIII, and then cloned into pMV261, yielding pMV261-NudC_Sm_. The *inhA* and *Eth*A genes of *M. tuberculosis* H37Rv were amplified using primers InhANcoIFP/InhAHindIIIRP and EthABamHIFP/EthAHindIIIRP (Table S3), inserted into the NcoI/HindIII sites of pET28a and BamHI/HindIII sites of pET32a, creating pET28a-InhA and pET32a-EthA respectively.

### Protein expression and purification

Recombinant strains *E. coli* BL21 (DE3)/pET32a-NudC_BCG_, *E. coli* BL21 (DE3)/pET32a-NudC_Rv_ and *E. coli* BL21 (DE3)/pET28a-InhA were all induced with 0.5 mM isopropyl β-d-thiogalactopyranoside (IPTG) at 25°C for 10 h. Bacterial cells were harvested, suspended in binding buffer (50 mM Tris·HCl, 0.5 M NaCl, 25 mM imidazole, pH 8.0) and disrupted by sonication. All proteins were purified by nickel affinity chromatography. NudC protein used for analytical ultracentrifugation was then purified by gel filtration with a Superdex 200 10/300GL column (Pharmacia BioTech) which had previously been equilibrated with 50 mM Tris-HCl buffer (pH 8.0). The enzyme was eluted with 50 mM Tris-HCl buffer (pH 8.0), and peak fractions containing the enzyme were pooled.

### Analytical ultracentrifugation

The molecular weight of recombinant NudC was analysed using an XL-I analytical ultracentrifuge (Beckman Coulter, Fullerton, CA, USA) equipped with a four-cell An-60 Tirotor. Purified NudC (0.8 mg ml^−1^ in 50 mM Tris·HCl, pH 8.0) was centrifuged at 4°C under 40000 rpm for 4 h, using 50 mM Tris-HCl buffer (pH 8.0) as a control. After ultracentrifugation, data were analysed using SEDFIT (Schuck, 2000; http://www.analyticalultracentrifugation.com/download.htm).

### NudC activity assays

NudC activity was quantified by measuring the conversion of the phosphatase-insensitive substrate NADH to phosphatase-sensitive products AMP and NMNH (Xu *et al*., 2000). The standard reaction mixture (50 µl) contained: 50 mM Tris-HCl, pH 8.0; 5 mM MgCl_2_; 2.5 mM NADH; 4 units of calf intestine alkaline phosphatase (EC 3.1.3.1) and 0.2–2 milliunits of enzyme. After incubation at 37°C for 15 min, the reaction was terminated by addition of 250 µl of 4 mM EDTA. Inorganic orthophosphate was measured as previously reported (Ames and Dubin, 1960). A unit of enzyme catalysed the hydrolysis of 1 µmol of NADH per min under these conditions.

### Synthesis and isolation of INH-NAD and ETH-NAD adducts

First, the [Mn^III^(H_2_P_2_O_7_)_3_]Na_3_ complex which is necessary for synthesis of the INH-NAD adduct was synthesized as previously described (Nguyen *et al*., 2001). In brief, an aqueous solution of sodium pyrophosphate (200 mM) containing Mn^III^(OAc)_3_ (5.5 mM) was acidified to pH 4.5 (or pH 6.5) with pyrophosphoric acid, then stirred at RT for 24 h. The INH-NAD adduct was then synthesized as reported (Nguyen *et al*., 2002). The reaction mixture containing 100 mM phosphate buffer (pH 7.5), 2 mM INH, 2 mM NAD and 4 mM Mn(III) pyrophosphate (introduced in 10 consecutive additions of 400 µM each, every 2 min) was stirred at RT for 20 min after addition of the last ingredient. To isolate the INH-NAD adduct, purified InhA (400 µM) was added into the above reaction mixture and stirred at RT for 2 h. The sample was then concentrated and the buffer was exchanged with 50 mM Tris-HCl by using a Millipore Amicon Ultra 10 K centrifugal device. The InhA-adduct complex was denatured by boiling for 40 s and immediately cooling on ice and then transferred into a Microcon 3 centricon filter unit and spun to recover the released adduct in the filtrate (Lei *et al*., 2000). The ETH-NAD adduct was synthesized as previously described (Wang *et al*., 2007). Briefly, the plasmids pET28a-InhA and pET32a-EthA were co-transformed into *E. coli* BL21 (DE3). The strain containing these two plasmids was cultured in LB media containing 50 µg ml^−1^ kanamycin and 100 µg ml^−1^ ampicillin at 37°C until OD_600_ reached 0.8, then induced at 16°C for 20 h by addition of 0.5 mM IPTG. ETH of 100 µg ml^−1^ was also added to the culture during induction. Recombinant InhA was purified according to the above method. The InhA purified from the strains containing both pET28a-InhA and pET32a-EthA plasmids was concentrated and heated for 40 s at 100°C. After heat treatment, ETH-NAD was separated from the denatured InhA by filtration using a Microcon 3 centricon filter unit.

### HPLC-MS analysis of INH-NAD and ETH-NAD adducts and their NudC hydrolysis products

The INH-NAD adduct and its NudC hydrolysis products were analysed by HPLC-ESI/MS (Shimadzu LCMS-2010EV, Tokyo, Japan). HPLC separations were performed according to Rawat (Rawat *et al*., 2003). A Shim-pack VP-ODS column (Shimadzu, 250 × 2.0 mm i.d., 5 µm) fitted with a C18 guard column (Shimadzu) was used. ESI/MS working parameters were as follows: capillary voltage was 4.5 kV, curved desolvation line (CDL) was 250, and heat block temperatures for the analysis was set at 200°C. Nitrogen drying and nebulizer gases were set at 1.5 l min^−1^ with a pressure of 0.02 MPa. The detector voltage was set at 1.4 eV. The ETH-NAD adduct and its NudC hydrolysis products were analysed on a HPLC-MicrOTOF_Q mass spectrometer (Agilent 1200 HPLC and Bruker Daltonic MicrOTOF_Q mass spectrometer). HPLC separations were carried out under the same conditions as those for INH-NAD. The ESI/MS working parameters were as follows: the ESI source was operated with a nebulizer pressure of 0.8 bar while the drying gas was delivered at a flow rate of 8 l min^−1^ and the capillary voltage was 4000 V. All MS data were acquired in a scan range between 200 and 1000 m/z under the negative ionization mode.

### Construction of mycobacterial mutant strains

Temperature-sensitive specialized transducing phasmids (Bardarov *et al*., 2002) were constructed and used for allelic exchange-mediated deletion of the *nudC* gene in different mycobacteria (MSMEG_1946, Rv3199c/MRA_3236 and BCG_3224c). DNA segments of approximately 1 kb flanking the gene of interest were amplified by PCR, digested with Van91I and ligated to compatible fragments of the counter-selectable vector p0004S (a kind gift from Dr William R. Jacobs Jr). Allelic exchange plasmids were selected and propagated following transformation of *E. coli* TOP10 to hygromycin resistance. Sequences of inserted DNA fragments were verified. Allelic exchange plasmids were digested with PacI and ligated to PacI-digested phAE159 (Lee *et al*., 2004). Ligation products were packaged using MaxPlax™ Lambda Packaging Extracts (EPICENTRE Biotechnologies, USA) and transduced into *E. coli* HB101 for propagation as cosmids. Cosmid DNA was purified and electroporated into *M. smegmatis* mc^2^155 for phage propagation at the permissive temperature (30°C). Subsequently, *M. smegmatis* mc^2^155 strains were transduced at the non-permissive temperature (37°C) and recombinant strains with integration of the allelic exchange marker were selected on LB agar plates containing hygromycin. Integration of the allelic exchange marker at the locus of interest was verified by PCR using one primer that anneals within the exchange marker and one primer that anneals distal to the genomic region that was involved in allelic exchange.

### Drug susceptibility tests

Mycobacterial cells were grown to mid-log phase (OD_600_ of 0.5–1.0) and diluted to 10^6^ cfu ml^−1^ (cfu, colony-forming unit) in fresh 7H9 medium. Then 10-fold serial dilutions were plated onto 7H10 agar solid plates containing various concentrations of different drugs: for *M. smegmatis* mc^2^155 strains, INH (0, 0.1, 0.25, 0.5, 1.25, 2.5, 5, 10, 25, 50, 100, 150, 200 µg ml^−1^), ETH (0, 0.1, 0.25, 0.5, 1.25, 2.5, 5, 10, 25, 50, 100, 150, 200 µg ml^−1^), EMB (0, 0.1, 0.25, 0.5, 1, 2.5, 5 µg ml^−1^) and RIF (0, 5, 10, 25, 50, 75, 100 µg ml^−1^); for *M. bovis* BCG and *M. tuberculosis* H37Ra strains, INH (0, 0.1, 0.2, 0.3, 0.4, 0.5, 0.6, 0.8, 1, 2 µg ml^−1^), ETH (0, 2.5, 5, 10, 15, 20, 50 µg ml^−1^), EMB (0, 0.1, 0.25, 0.5, 1, 2.5, 5, 10 µg ml^−1^) and RIF (0, 0.005, 0.01, 0.05, 0.1, 0.25, 0.5 µg ml^−1^). Cultures were incubated for 3 days (for *M. smegmatis* mc^2^155) or 21 days (for *M. bovis* BCG and *M. tuberculosis* H37Ra) at 37°C. MIC was defined as the lowest concentration of compound required to inhibit 99% of bacterial cfu.

### Drug exposure experiments

Mycobacterial cells were grown to mid-log phase (OD_600_ of 0.5–1.0) and diluted to about 10^6^–10^7^ cfu ml^−1^ in fresh 7H9 medium with or without 10% OADC. For INH treatment of *M. smegmatis* mc^2^155, 50 µg ml^−1^ INH were added to each sample, for that of *M. bovis* BCG, 5 µg ml^−1^ INH was added to each sample. For ETH treatment of *M. bovis* BCG, 50 µg ml^−1^ ETH was added to each sample. Aliquots of samples were taken at regular intervals and serial dilutions were performed before plating.

### Analysis of mycobacterial mycolic acids following ETH and INH treatment

Extraction of cell wall mycolic acids from mycobacterial cells was carried out as previously described (Baulard *et al*., 2000). *M. smegmatis* mc^2^155 or *M. bovis* BCG cultures (5 ml) were grown to mid-log phase (OD_600_ ≍ 0.3), then ETH or INH was added at various concentrations followed by further incubation for 4 h (for *M. smegmatis* mc^2^155) or 24 h (for *M. bovis* BCG). At this point, 1, 2-[^14^C] acetate (1 µCi ml^−1^) was added and the cultures were further incubated with gentle agitation at 37°C for 4 h (for *M. smegmatis* mc^2^155) or 24 h (for *M. bovis* BCG). After centrifugation, cell pellets were washed once with distilled water and then resuspended in 3 ml of 15% tetrabutylammonium hydroxide (TBAH). The cell suspension was heated at 100°C overnight. After cooling, water (2 ml), dichloromethane (1 ml) and iodomethane (250 µl) were added, and the entire reaction mixture was agitated for 30 min. After centrifugation, the upper aqueous phase was discarded and the lower organic phase was washed twice with water, dried in a sand bath, and the residue was resuspended in methylene chloride. The radiolabelled extracts were separated by thin-layer chromatography (TLC) on a silica gel 60 *F*_254_ plate and developed once in petroleum ether: acetone (95:5, v/v). Subsequent autoradiography revealed ^14^C-labelled FAMEs and MAMEs after overnight exposure of the TLC plates to Kodak BioMax MR film.
