# Supplementary material for: Comparative analysis of mycobacterial NADH pyrophosphatase isoforms reveals a novel mechanism for isoniazid and ethionamide inactivation
Source: Mol Microbiol. 2011 Nov 3;82(6):1375–91. doi: 10.1111/j.1365-2958.2011.07892.x (PMC3283747; doi:10.1111/j.1365-2958.2011.07892.x)
Supplement: Supplementary file 1 [file mmi0082-1375-SD1.pdf]

Supplementary Experimental Procedures

Preparation of anti-NudC antibodies

A polyclonal antibody against NudC was raised by immunization of rabbits with purified $NudC_{Rv}$ and $NudC_{BCG}$ protein. NudC antibodies were purified from rabbit serum by affinity chromatography using protein A beads (Beyotime Institute of Biotechnology, Beijing, China) with glycine-HCl as elutant, dialysed against phosphate-buffered saline. The titre of the purified antibodies was determined by ELISA.

Western blotting

Samples were separated by SDS-PAGE (12-15% acrylamide) and then transferred to PVDF membranes using a BioRad SD device (BioRad Laboratories) (20-30 min at 15 V). The membrane was blocked overnight at 4°C in 1×TBST (Tris-buffered saline plus 0.1% Tween-20) containing 5% NFDM (non-fat dry milk). Primary rabbit anti-NudC (1:5000) polyclonal antibodies were diluted in TBST/1% NFDM and incubated at 37°C for 2 h. After washing with TBST, the membrane was incubated with alkaline phosphatase-conjugated goat anti-rabbit antibody (1:5000 dilution) for 1 h at 37°C, then detected according to the manufacturer's instructions.

Transformation of mycobacteria

Mycobacterial competent cells were prepared as previously reported (Larsen, 2000). Transformations were performed using a BTX ECM 630 electroporator (2.5 kV, 25 μF, and 1,000 Ω).

References

Larsen, M.H. (2000) Some common methods in mycobacterial genetics. In *Molecular genetics of mycobacteria*. Hatfull, G.F., and Jacobs, W.R., Jr (eds). Washington, D C: American Society for Microbiology Press, pp. 313-320.

Table S1. Sequencing and analysis of *nudC* genes from 137 *M. tuberculosis* clinical isolates from China*

Mutation position	Nucleotide change	Amino acid change	No.(%) of samples
Pro (237)	CCG→CAG	Pro→Gln	29 (21.2)
Pro (237),<br>Pro (239)	CCG→CAG,<br>CCG→CGG	Pro→Gln,<br>Pro→Arg	103 (75.2)
Ala (130),<br>Pro (237),<br>Pro (239)	GCG→ACG,<br>CCG→CAG,<br>CCG→CGG	Ala→Thr,<br>Pro→Gln,<br>Pro→Arg	4 (2.9)
Ala (146),<br>Pro (237),<br>Pro (239)	GCG→GCA,<br>CCG→CAG,<br>CCG→CGG	Ala→Ala,<br>Pro→Gln,<br>Pro→Arg	1 (0.7)

*All *nudC* genes from *M. tuberculosis* clinical isolates were compared to the *nudC* gene of *M. bovis* BCG.

Table S2. Substrate specificity of $NudC_{Rv}$ and $NudC_{BCG}$

	Relative activity (%)	
	M. tuberculosis H37Rv	*M. bovis* BCG
NADH	20	100
NAD^+	3	40
NADPH	5	44
$NADP^+$	0	20
ADP-ribose	0	12

All substrates were tested at a concentration of 2.5 mM, in the presence of Mn^{2+}.

Table S3. Plasmids, strains and primers used in this study

Category	Name	Relevant Features/Sequences	Source
Plasmids			
	pET32a-NudC$_{BCG}$	pET32a with *M. bovis* BCG *nudC* inserted downstream of the T7 promoter, Ap^R	This study
	pET32a-NudC$_{Rv}$	pET32a with *M. tuberculosis* H37Rv *nudC* inserted downstream of the T7 promoter, Ap^R	This study
	pET28a-InhA	pET28a with *M. tuberculosis* H37Rv *inhA* inserted downstream of the T7 promoter, Km^R	This study
	pET32a-EthA	pET32a with *M. tuberculosis* H37Rv *EthA* inserted downstream of the T7 promoter, Ap^R	This study
	pMV261-NudC$_{BCG}$	pMV261 with *M. bovis* BCG *nudC* inserted downstream of the hsp60 promoter, Km^R	This study
	pMV261-NudC$_{Rv}$	pMV261 with *M. tuberculosis* H37Rv *nudC* inserted downstream of the hsp60 promoter, Km^R	This study
	pMV261-NudC$_{Sm}$	pMV261 with *M. smegmatis* mc^{2}155 *nudC* inserted downstream of the hsp60 promoter, Km^R	This study
Strains			
	mc^{2}155 pMV261	*M. smegmatis* mc^{2}155 transformed with pMV261	This study
	mc^{2}155 pMV261::*nudC*$_{BCG}$	*M. smegmatis* mc^{2}155 transformed with pMV261-NudC$_{BCG}$	This study
	mc^{2}155 pMV261::*nudC*$_{Rv}$	*M. smegmatis* mc^{2}155 transformed with pMV261-NudC$_{Rv}$	This study
	BCG pMV261::*nudC*$_{BCG}$	*M. bovis* BCG transformed with pMV261-NudC$_{BCG}$	This study
	BCG pMV261::*nudC*$_{Rv}$	*M. bovis* BCG transformed with pMV261-NudC$_{Rv}$	This study
	BCG pMV261	*M. bovis* BCG transformed with pMV261	This study
	mc^{2}155 Δ*nudC*	Specialized transduction of strain *M. smegmatis* mc^{2}155 with phAES*nudC*$_{Sm}$	This study
	mc^{2}155 pMV261::*nudC*$_{Sm}$	*M. smegmatis* mc^{2}155 transformed with pMV261-NudC$_{Sm}$	This study
	BCG Δ*nudC*	Specialized transduction of strain *M. bovis* BCG with phAES*nudC*$_{BCG}$	This study
	H37Ra Δ*nudC*	Specialized transduction of strain *M. tuberculosis* H37Ra with phAES$_{Ra}$	This study
	mc^{2}155 Δ*nudC* pMV261::*nudC*$_{Sm}$	mc^{2}155 Δ*nudC* transformed with pMV261-NudC$_{Sm}$	This study
	BCG Δ*nudC* pMV261::*nudC*$_{BCG}$	BCG Δ*nudC* transformed with pMV261-NudC$_{BCG}$	This study
Primers*			

NudC*Nco*IFP	5'-GTCACCATGGCTATGACGAACGTAAGCGGCGTGGAT-3'	This study
NudC*Xho*IRP	5'-TGCACTCGAGTTATCATTCGCACGCTGCCCACG-3'	This study
261NudC*Bam*HIFP	5'-ATCGGGATCCATGACGAACGTAAGCGGCG-3'	This study
261NudC*Hin*dШRP	5'-TGCAAAGCTTTCATTCGCACGCTGCCCACG-3'	This study
261NudC*Bam*HIFP$_{Sm}$	5'-ATCGGGATCCATGAGCGAACACCGCACGT-3'	This study
261NudC*Hin*dШRP$_{Sm}$	5'-TGCAAAGCTTTCAGTCGAGTGCGGCCCAGG-3'	This study
InhA*Nco*IFP	5'-ACAACCATGGGAATGACAGGACTGCTGGACGGC-3'	This study
InhA*Hin*dШRP	5'-ATGCAAGCTTGAGCAATTGGGTGTGCGCGCCG-3'	This study
MSNudCLFP	5'-TTTTTTTTCCATAAATTGGATGCCACGAAATCCGACATCCT-3'	This study
MSNudCLRP	5'-TTTTTTTTCCATTTCTTGGTTGGCGATGAGCACCTGGTT-3'	This study
MSNudCRFP	5'-TTTTTTTTCCATAGATTGGTGCTCAACTGGCATGACAACGC-3'	This study
MSNudCRRP	5'-TTTTTTTTCCATCTTTTGGAACCCGACCATCAAGCAGGTCAA-3'	This study
LCNudCFP	5'-AACGTGAGGTGGAGCAGGCCGAGGT-3'	This study
LCNudCRP	5'-AGCGCGGACGAGGTCAAAGCGAAGC-3'	This study
LBRnudcFP	5'-TTTTTTTTCCATAAATTGGTCGGCTGACGTTTCTGGATTG-3'	This study
LBRnudcRP	5'-TTTTTTTTCCATTTCTTGGGAAAATCCACGCCGCTTACG-3'	This study
RBRnudcFP	5'-TTTTTTTTCCATAGATTGGATCGAATCGTGGGCAGCGTG-3'	This study
RBRnudcRP	5'-TTTTTTTTCCATCTTTTGGGAAAGGTCAGCGCCTGCACAG-3'	This study
EthA*Bam*HIFP	5'-CTGGGGATCCACCGAGCACCTCGACGT-3'	This study
EthA*Hin*dШRP	5'-GCTAAAGCTTTTAAACCCCCACCGGGGCA-3'	This study

* The underlined sequences indicate restriction sites

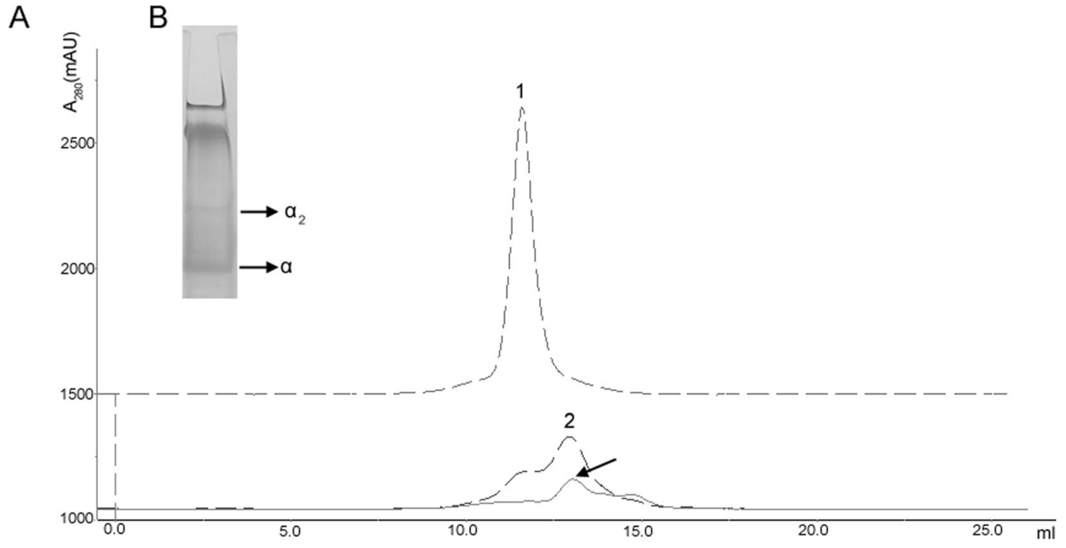

Fig. S1. Analytical ultracentrifugation and native PAGE analysis of the native structure of $NudC_{QR}$. A. Gel exclusion chromatography analysis of the native structure of purified $NudC_{QR}$. Nickel affinity purified $NudC_{QR}$ was concentrated then loaded onto a Superdex 200 10/300GL column and eluted. The $NudC_{QR}$ elution peak is indicated by an arrow. The broken line indicates $NudC_{BCG}$ (peak 1 indicated) and $NudC_{Rv}$ (peak 2 indicated) eluted separately. B. Native PAGE analysis of purified $NudC_{QR}$ on 9% (v/v) PAGE. Two bands of $NudC_{QR}$ (α_2 and α) were present on native PAGE (indicated by arrows).

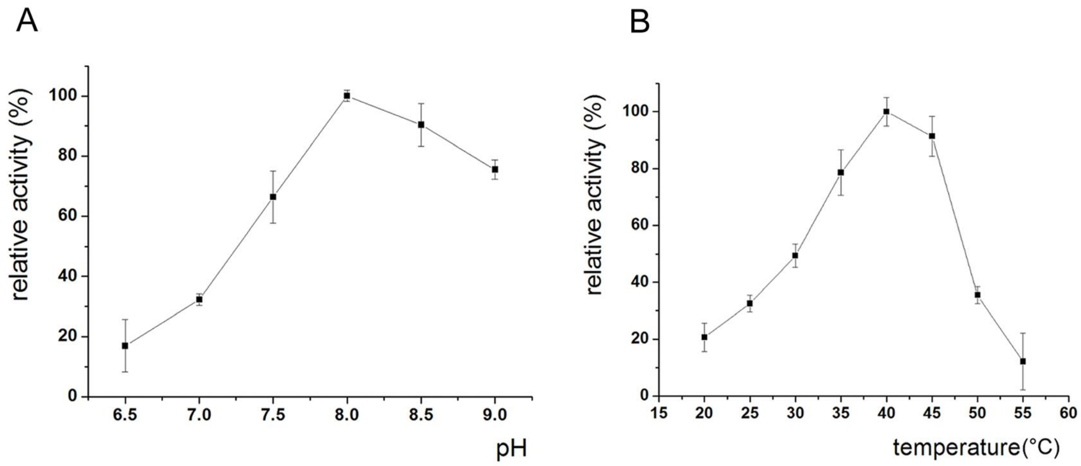

Fig. S2. The effect of pH and temperature on $NudC_{BCG}$ enzyme activity. Activities were determined using NADH as the substrate in the presence of Mg^{2+}. A. The effect of pH on $NudC_{BCG}$ enzyme activity. The buffer used was 50 mM Tris·HCl, adjusted to various pH levels. B. The effect of temperature on $NudC_{BCG}$ enzyme activity. The activity of $NudC_{BCG}$ was obtained at various temperature ranges using 50 mM Tris·HCl buffer (pH 8.0) as the solvent. The experiment was performed in triplicate. Error bars indicate standard deviations.

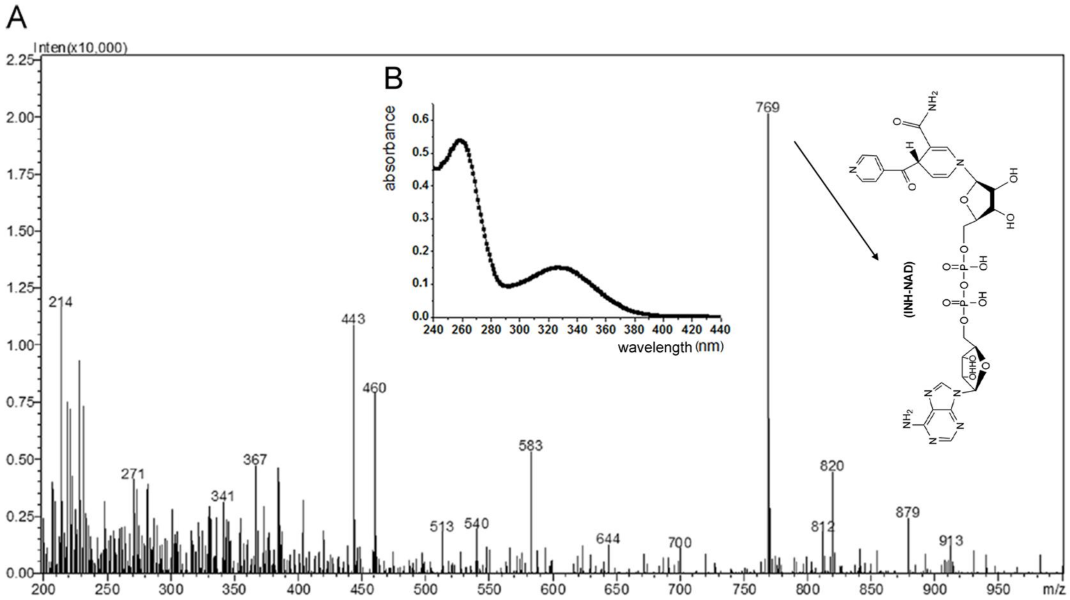

Fig. S3. HPLC-MS and spectrometric verification of the synthesized INH-NAD adduct. A. MS analysis of the synthesized INH-NAD adduct. The product peak is indicated by an arrow, and the chemical structure of the product is shown nearby. INH-NAD, calculated weight = 770 and found weight = 769 [(M-H)$^-$] (negative mode). MS data was acquired in the negative mode. B. Spectrometric characterizations of the synthesized INH-NAD adduct.

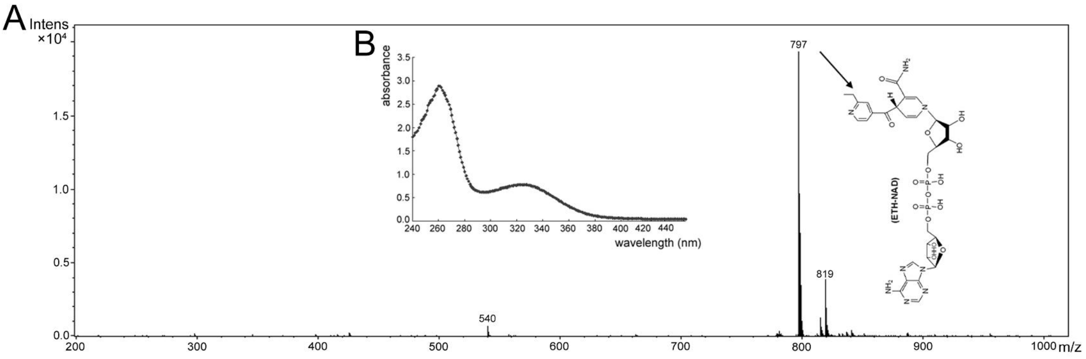

Fig. S4. HPLC-MS and spectrometric verification of the synthesized ETH-NAD adduct. A. MS analysis of the synthesized ETH-NAD adduct. The product peak iss indicated by an arrow, and the chemical structure of the product is shown nearby. ETH-NAD, calculated weight = 798 and found weight = 797 [(M-H)$^-$] (negative mode). MS data was acquired in the negative mode. B. Spectrometric characterizations of the synthesized ETH-NAD adduct.

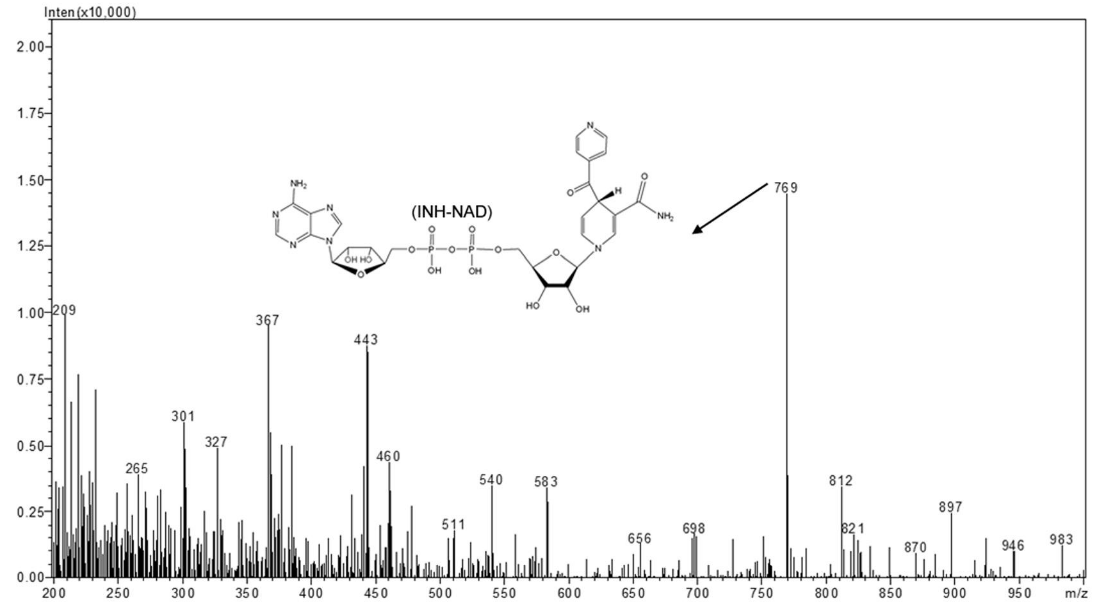

Fig. S5. HPLC-MS analysis of the INH-NAD adduct hydrolyzed by $NudC_{QR}$. Purified $NudC_{QR}$ was incubated with the synthesized INH-NAD adduct in the presence of Mg^{2+}, and reacted sufficiently. The reaction mixture was then filtered to remove the protein and the filtrate was analyzed by HPLC-MS. The product peak in the MS spectra is indicated by an arrow, and the chemical structure of the product is shown nearby. INH-NAD, calculated weight = 770 and found weight = 769 $[(M-H)^-]$ (negative mode). MS data was acquired in the negative mode.

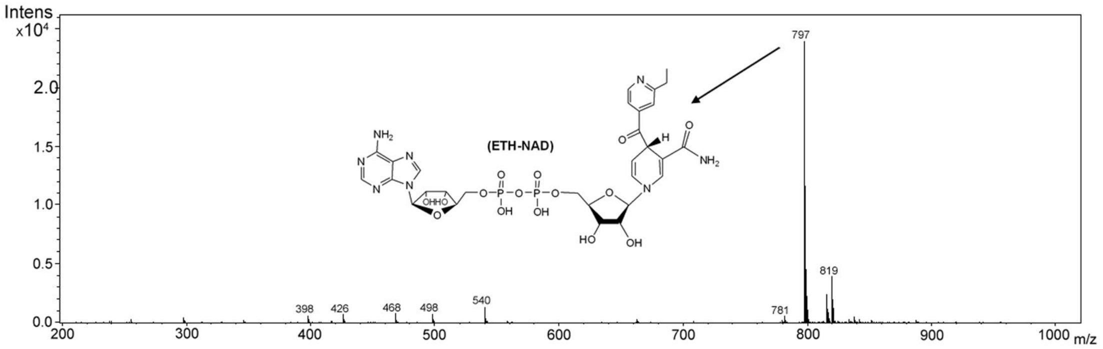

Fig. S6. HPLC-MS analysis of the ETH-NAD adduct hydrolyzed by NudC$_{QR}$. Purified NudC$_{QR}$ was incubated with the synthesized ETH-NAD adduct in the presence of Mg^{2+}, and reacted sufficiently. The reaction mixture was then filtered to remove the protein and the filtrate was analyzed by HPLC-MS. The product peak in the MS spectra is indicated by an arrow and the chemical structure of the product is shown nearby. ETH-NAD, calculated weight = 798 and found weight = 797 [(M-H)$^-$] (negative mode). MS data was acquired in the negative mode.

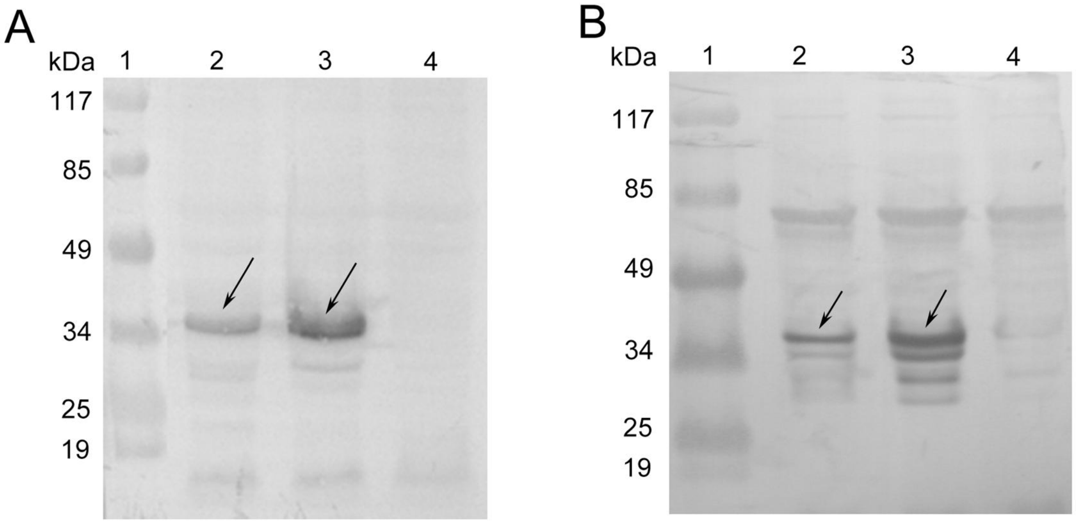

Fig. S7. Verification of NudC over-expression in *M. smegmatis* mc^{2}155. Western blotting using anti-NudC$_{Rv}$ (A) or anti-NudC$_{BCG}$ (B) antibody. Lane 1, protein molecular weight marker; lanes 2-4, cell-free extracts of *M. smegmatis* mc^{2}155 carrying pMV261-NudC$_{Rv}$, pMV261-NudC$_{BCG}$ and pMV261. The arrows indicate the expected products.

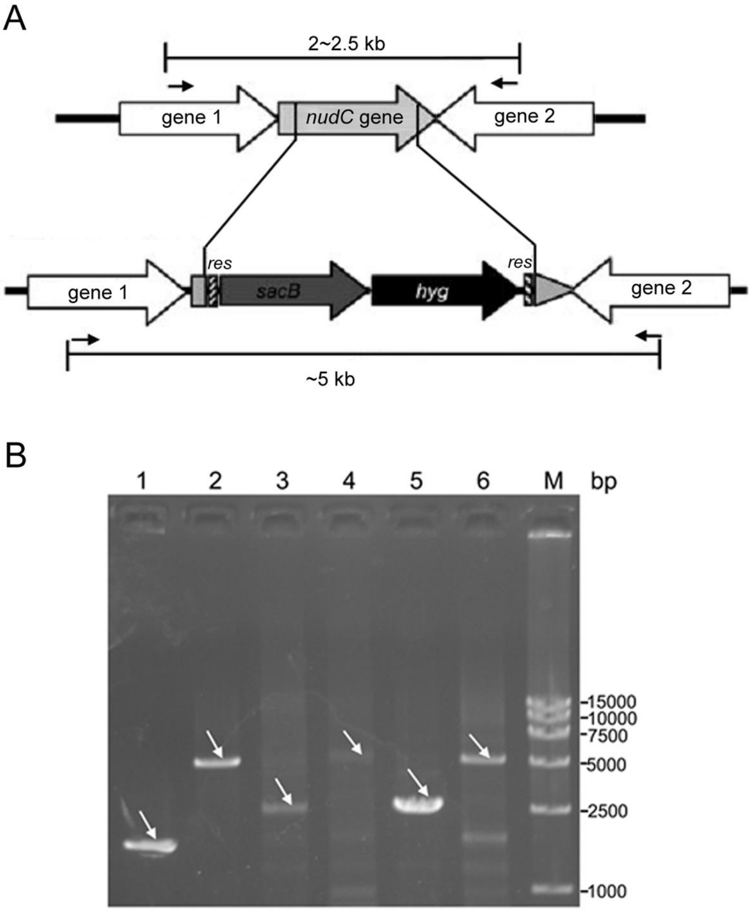

Fig. S8. Map of the *nudC* gene knock-out and PCR verification. A, Map of the *nudC* gene region in the genome and its corresponding region in the conditional mutant strain. Gene 1/gene 2 indicate the up/downstream gene regions of the *nudC* gene; *res*, γδ resolvase site; *hyg*, hygromycin resistance gene; *sacB*, sucrose counterselectable gene. The primers used for PCR amplification are indicated by arrows and the expected sizes of the PCR products are indicated by straight lines. B, PCR verification of *nudc* gene knock-out strains. Agarose gel electrophoresis was performed to verify the PCR products amplified from genomic DNA of different mycobacterial strains. Lane 1, *M. smegmatis* mc^2155; Lane 2, mc^2155 Δ*nudC*; Lane 3, *M. bovis* BCG; Lane 4, BCG Δ*nudC*; Lane 5, *M. tuberculosis* H37Ra; Lane 6, H37Ra Δ*nudC*; M, DNA marker. PCR products are indicated by white arrows.

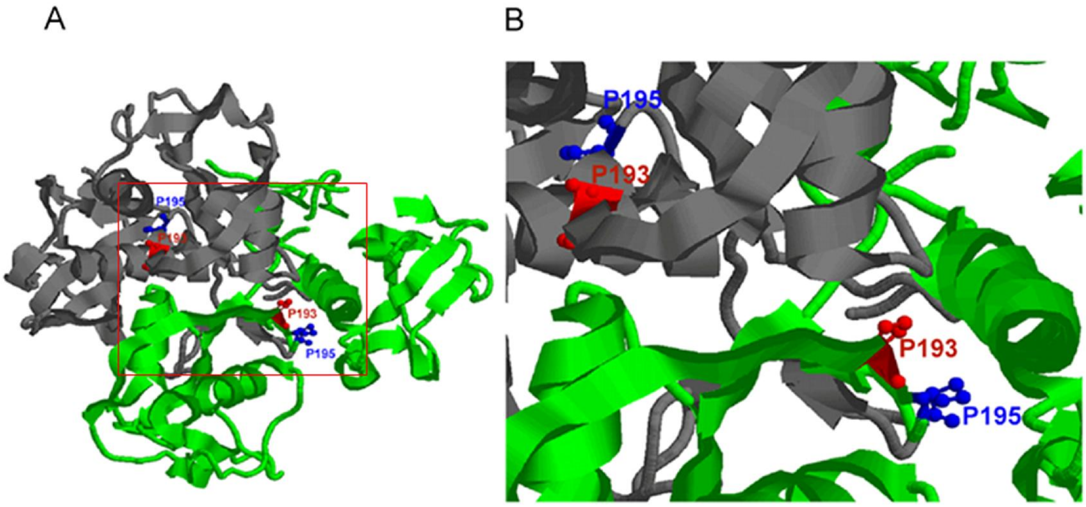

Fig. S9. Cartoon diagram of the structure of *E. coli* NudC. A. *E. coli* NudC is a dimeric protein. Chains a and b are colored in green and grey, respectively. Residues P193 and P195 are shown as ball and stick models, colored red and blue, respectively. B. Enlargement of the red box in A.
